# Clinical Value of a Novel Apparent Diffusion Coefficient-Based Bi-Color Map for Detecting Clinically Significant Prostate Cancer: A Retrospective Study

**DOI:** 10.3390/cancers18111796

**Published:** 2026-06-01

**Authors:** Mitsuo Okada, Yoichi Araki, Yosuke Hirasawa, Go Nagao, Takeshi Kashima, Kenjiro Hayashi, Naoya Satake, Kazuhiro Saito, Yoshio Ohno

**Affiliations:** 1 Department of Urology, Tokyo Medical University, 6-7-1 Nishishinjuku, Shinjuku-ku, Tokyo 1600023, Japan; m_okada@tokyo-med.ac.jp (M.O.);; 2 Department of Radiology, Tokyo Medical University, 6-7-1 Nishishinjuku, Shinjuku-ku, Tokyo 1600023, Japan; araki@tokyo-med.ac.jp (Y.A.); saito-k@tokyo-med.ac.jp (K.S.)

**Keywords:** prostatic neoplasms, magnetic resonance imaging, diffusion magnetic resonance imaging, diagnosis, retrospective studies

## Abstract

**Background:** Minimum apparent diffusion coefficient (ADC) values are independent predictors of clinically significant prostate cancer (csPC). We developed a novel ADC-based bi-color map and evaluated its utility for identifying lesions suspicious for csPC. **Methods:** We retrospectively analyzed 108 targeted prostate biopsy cases and 93 radical prostatectomy cases. In the biopsy cohort, we assessed the association between ADC-based bi-color map positivity and csPC in lesions with a Prostate Imaging–Reporting and Data System (PI-RADS) score ≥ 3. In the prostatectomy cohort, we evaluated additional color map-positive lesions not categorized as PI-RADS ≥ 3 on preoperative MRI. **Results:** In the biopsy cohort, 118 lesions were positive on the ADC-based bi-color map, including lesions outside PI-RADS ≥ 3 categories. Among 157 lesions with a PI-RADS score ≥ 3, csPC was detected in 65 lesions (41.4%). Of these lesions, 70 (44.6%) were positive on the bi-color map, and csPC was identified in 49 (70.0%). The added value of the bi-color map was most evident in PI-RADS 4 lesions, where csPC detection rates were significantly higher in color map-positive than in color map-negative lesions (74.4% vs. 23.3%). In the prostatectomy cohort, 215 lesions were positive on the bi-color map, and csPC was confirmed pathologically in 126 lesions (58.6%). Among 82 color map-positive lesions not classified as PI-RADS ≥3, 55 (67.1%) corresponded to csPC. **Conclusions:** The ADC-based bi-color map may improve lesion highlighting and risk stratification for csPC and help identify suspicious lesions overlooked on conventional MRI assessment.

## Simple Summary

Prostate biopsy is commonly performed following prostate-specific antigen (PSA) screening; however, overdiagnosis and overtreatment of clinically insignificant prostate cancer remain major clinical concerns. To improve patient selection for biopsy, multiparametric magnetic resonance imaging (MRI) is increasingly used before biopsy, and biopsy decisions are often based on the Prostate Imaging–Reporting and Data System (PI-RADS). Although the PI-RADS is useful for detecting clinically significant prostate cancer (csPC), its interpretation is subjective and strongly influenced by reader experience, resulting in potential false-positive and false-negative findings. The apparent diffusion coefficient (ADC), derived from diffusion-weighted MRI, has been reported to correlate with tumor presence and aggressiveness. Therefore, we developed a novel ADC-based bi-color map that objectively analyzes MRI data using fixed ADC thresholds to automatically highlight lesions suspicious for csPC. This approach may improve lesion detection, support risk stratification, and complement conventional MRI interpretation in clinical practice.

## 1. Introduction

Prostate cancer (PC) is the second most commonly diagnosed cancer in men and the fifth leading cause of cancer-related deaths. In 2020, an estimated 1.4 million new cases of PC occurred worldwide, with a total of 375,000 deaths [1].

Multiparametric magnetic resonance imaging (MRI) has been reported to enhance the diagnostic accuracy for PC [2,3]. In addition, the Prostate Imaging–Reporting and Data System (PI-RADS) was developed for standardization of the acquisition and interpretation of MRI findings of PC. PI-RADS v2, which was released in 2015, improved the detection rate of clinically significant prostate cancer (csPC) [4,5,6]. However, the positivity rates of each PI-RADS score differed among several reports [7,8,9,10,11], with concerns such as inter-reader variability [12,13]. Meanwhile, some studies have reported that a lower Apparent Diffusion Coefficient (ADC) value on MRI is strongly correlated with the presence of csPC in suspected lesions [14,15,16,17,18]. In recent reports on cut-off values of ADC for predicting csPC [14,15,16,17], the cut-off values of the mean ADC ranged from 750 µm^2^/s to 830 µm^2^/s. Wu et al. reported that a minimum ADC of <570 µm^2^/s could differentiate between Gleason scores of 3 + 4 and 3 + 3 [18]. We have previously analyzed the association between ADC values and the presence of csPC in the transitional zone of lesions in patients with a PI-RADS score of 4 or 5. We finally demonstrated that the minimum ADC value was an independent predictor of csPC and that a threshold of ADC < 595 µm^2^/s significantly improved the detection rate for csPC compared with that of using the PI-RADS score alone [19].

Recently, to translate these findings into clinical practice, we developed a novel bi-color map based on maximum and minimum ADC values. In this study, we aimed to evaluate the clinical value of this bi-color map.

## 2. Materials and Methods

This retrospective study was conducted in accordance with the ethical guidelines for clinical studies of the Ministry of Health, Labor, and Welfare of Japan and was approved by the Ethics Committee of Tokyo Medical University (approval number: T2023-0158). We had provided a public notice on our website regarding explanatory consent and the opportunity to refuse. Therefore, the need for informed consent was waived by the Ethics Committee of Tokyo Medical University. We retrospectively reviewed the clinical data of 108 patients with PI-RADS score ≥ 3 lesions who underwent MRI-transrectal ultrasound (TRUS) fusion imaging-guided biopsy for suspected lesions of PC between October 2022 and July 2023 and of 93 patients who underwent prostate MRI and robot-assisted radical prostatectomy (RARP) at our institution between 2018 and 2022. MRI was performed using a 3.0 Tesla scanner with a 60-channel coil system (Skyra, Siemens, Erlangen, Germany). A unified protocol was used for all MRI examinations. Axial, coronal, and sagittal T2-weighted images were obtained using the following parameters: repetition time (TR), 3000–5480 ms; echo time (TE), 86–107 ms; flip angle, 125–150°; slice thickness, 3 mm; and resolution, 0.4 × 0.4 mm. Axial diffusion-weighted imaging was performed, and the parameters were as follows: TR, 3720–7970 ms; TE, 57–81.18 ms; flip angle, 180°; b-values, 0, 800, 1500 s/mm^2^ or 0, 1000, 1500 s/mm^2^; average, 3; resolution, 0.83 × 0.83 mm. Quantitative ADC maps were created on a voxel-by-voxel basis for all b-values using the software on the scanner. Among patients who underwent MRI-TRUS fusion imaging-guided biopsy, 49 underwent MRI at external institutions. At an external institution, MRI was performed using a 1.5 or 3.0 Tesla system. Axial diffusion-weighted imaging was performed, and b-values ranging from 0 to 2000 were used in various combinations. ADC maps were generated from at least two b-values, including a low b-value (0–100 s/mm^2^) and a high b-value (800–2000 s/mm^2^), depending on scanner-specific protocols. MRI interpretations were performed during routine clinical practice using either PI-RADS version 2 or version 2.1 at the discretion of the interpreting radiologist. Because of the retrospective study design, the exact distribution of PI-RADS versions used in individual cases could not be reliably determined.

MRI-TRUS fusion imaging-guided prostate biopsy was performed using the Trinity^®^ system (Koelis, Meylan, France) via a transperineal approach. In principle, two or three cores were obtained by targeted biopsy of suspicious areas, and then 12 core systematic biopsies were obtained. csPC was uniformly defined as International Society of Urological Pathology (ISUP) grade group ≥2 in both the biopsy and prostatectomy cohorts.

**Figure 1:**
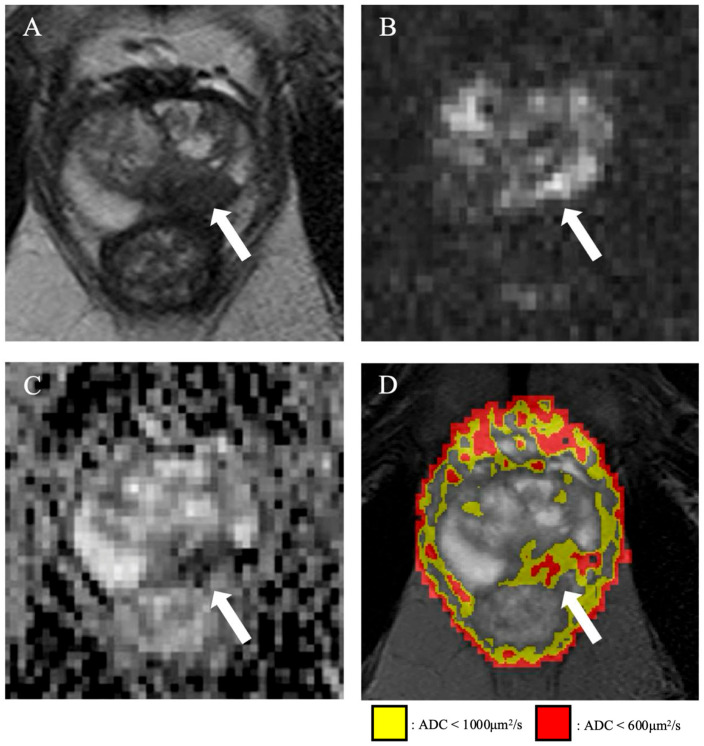
Bi-color map by the ADC value. (**A**) Hypointense lesion in the left peripheral zone on the T2-weighted image (arrow). (**B**) Hyperintense lesion on the high b-value diffusion-weighted image (arrow). (**C**) Hypointense lesion on the Apparent Diffusion Coefficient map (arrow). (**D**) The lesion with ADC < 1000 µm^2^/s and <600 µm^2^/s is presented in yellow and red, respectively. Lesions with ADC < 1000 µm^2^/s, including that with ADC < 600 µm^2^/s, are considered suspect lesions for clinically significant prostate cancer (arrow).

A bi-color map was automatically created by delineating lesions with ADC values < 1000 µm^2^/s in yellow and those with ADC values < 600 µm^2^/s in red on the axial ADC image. These two thresholds of 1000 µm^2^/s and 600 µm^2^/s were set based on a mean maximum ADC value of 922 μm^2^/s and the mean minimum ADC value of 595 μm^2^/s in our previous study [19]. Subsequently, these tracings were overlaid onto the corresponding axial T2-weighted images (Figure 1). A yellow lesion that includes the red area was defined as “positive: suspicious lesion of clinically significant prostate cancer.” Because this was a retrospective study involving multiple MRI platforms and acquisition protocols, no scanner-specific ADC normalization or phantom-based calibration was performed. A common ADC threshold was applied across institutions to evaluate the feasibility of a standardized bi-color mapping approach in a heterogeneous clinical setting.

**Figure 2:**
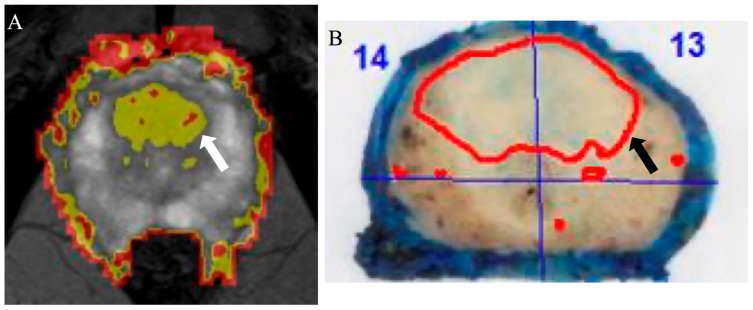
Association between positive areas on the bi-color map and the presence of clinically significant prostate cancer in radical prostatectomy specimen. (**A**) The color map presents the positive lesion in the transition zone (arrow). (**B**) Clinically significant cancer was detected in the corresponding lesion in the prostatectomy specimen (arrow).

First, we examined the positivity rate of the bi-color maps and explored the association between color map-positive lesions and the presence of csPC using the data of 108 patients with PI-RADS score ≥ 3 lesions who underwent MRI-TRUS fusion imaging-guided biopsy. Second, we analyzed the association between color map-positive lesions and the presence of csPC using the axial section with the largest tumor area in radical prostatectomy specimens (Figure 2). In radical prostatectomy specimens, distinct non-contiguous tumors were counted as separate lesions.

Chi-square tests were used to assess associations between categorical variables. The Cochran–Armitage trend test was used to evaluate trends across ordered PI-RADS categories. Diagnostic performance measures including sensitivity, specificity, positive predictive value, negative predictive value, and accuracy were calculated with 95% confidence intervals. Because several patients contributed more than one lesion, analyses were performed at the lesion level, and potential within-patient clustering was considered a methodological limitation. Statistical significance was set at *p* < 0.05, and SPSS Statistics software (version 31; IBM Corp., Armonk, NY, USA) was used for data management and analysis.

## 3. Results

### 3.1. Patient Characteristics

Table 1 shows detailed patient characteristics. In the group of patients who underwent MRI-TRUS fusion imaging-guided biopsy, the median age was 69.0 years (interquartile range [IQR]: 63.0‒74.0 years), and the median prostate-specific antigen (PSA) level was 7.70 ng/mL (IQR: 6.13‒11.10 ng/mL). Targeted biopsy was performed in 157 suspicious lesions with a PI-RADS score ≥ 3. In the group of patients who underwent RARP, the median age was 68.0 years (IQR: 63.0‒71.0 years), and the median PSA level was 7.7 ng/mL (IQR: 5.45‒13.0 ng/mL).

The results of targeted biopsies are shown in Table 2. The overall detection rate of csPC was 41.4% (65/157). This detection rate increased significantly with higher PI-RADS scores (PI-RADS score 3, 15.2%; PI-RADS score 4, 41.7%; and PI-RADS score 5, 81.0%; *p* < 0.001).

### 3.2. ADC-Based Bi-Color Map Results in Patients Who Underwent MRI-TRUS Fusion-Guided Biopsy

**Figure 3:**
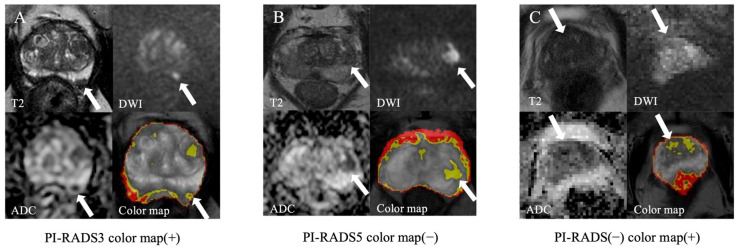
Typical examples of color maps by the PI-RADS score. (**A**) A small lesion in the left peripheral zone, which appeared hypointense on the axial T2-weighted image, was hyper- and hypointense on diffusion-weighted imaging and the apparent diffusion coefficient map, respectively, and was assigned a PI-RADS 3. This lesion was positive on the color map. (**B**) A large lesion in the left peripheral zone, which appeared hypointense on the axial T2-weighted image, being hyper- and hypointense on diffusion-weighted imaging and the apparent diffusion coefficient map, respectively, was assigned a PI-RADS 5. However, this lesion was negative on the color map and biopsy also yielded negative results for clinically significant prostate cancer. (**C**) This case highlights a color map-positive lesion, which was not suspicious for clinically significant cancer pre-biopsy MRI.

A total of 118 positive lesions were detected on the ADC-based bi-color map, and 48 lesions were not scored as PI-RADS score ≥ 3 before biopsy. Figure 3 shows representative examples of ADC-based bi-color maps according to PI-RADS category. Even for small lesions categorized as PI-RADS score 3, the areas showed positivity on the ADC-based bi-color map. Conversely, the ADC-based bi-color map-negative lesions were categorized as PI-RADS score 5 lesions. 

Table 3 shows the association between PI-RADS score and color map positivity. Seventy (44.6%) of one hundred fifty-seven lesions with PI-RADS score ≥ 3 were positive on the ADC-based bi-color map. The ADC-based bi-color map positivity was significantly associated with increasing PI-RADS score (*p* < 0.001).

Table 4 shows the association between ADC-based bi-color map positivity and csPC in the biopsy cohort. csPC was identified significantly more frequently in ADC-based bi-color map-positive lesions than in ADC-based bi-color map-negative lesions (70.0% vs. 18.4%, *p* < 0.001). Diagnostic performance analyses demonstrated that the ADC-based bi-color map had a sensitivity of 75.4% (95% confidence interval [CI], 63.1–85.2%), specificity of 77.2% (95% CI, 67.2–85.3%), positive predictive value of 70.0% (95% CI, 57.9–80.4%), negative predictive value of 81.6% (95% CI, 71.9–89.1%), and accuracy of 76.4% (95% CI, 69.0–82.8%) for csPC detection.

Table 5 shows biopsy results stratified by PI-RADS score and ADC-based bi-color map status. Among PI-RADS score 4 lesions, csPC was detected significantly more frequently in ADC-based bi-color map–positive lesions than in ADC-based bi-color map–negative lesions (74.4% vs. 23.3%, *p* < 0.001). In contrast, no statistically significant differences were observed in PI-RADS score 3 lesions (40.0% vs. 17.9%, *p* = 0.282) or PI-RADS score 5 lesions (88.2% vs. 50.0%, *p* = 0.148).

Subgroup analyses according to MRI acquisition site are summarized in Table 6. Significant associations between ADC-based bi-color map positivity and csPC were observed in both internally and externally acquired MRI examinations. In MRI examinations performed at our institution, the ADC-based bi-color map demonstrated a sensitivity of 79.5%, specificity of 58.3%, positive predictive value of 75.6%, negative predictive value of 63.6%, and accuracy of 71.4%. In MRI examinations performed at external institutions, the corresponding values were 69.2%, 83.8%, 62.1%, 87.7%, and 79.8%, respectively.

### 3.3. ADC-Based Bi-Color Map Results in Patients Who Underwent RARP

**Figure 4:**
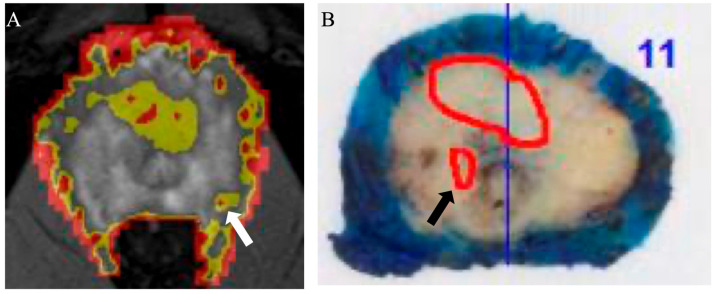
Typical examples of false-positive and false-negative cases in radical prostatectomy specimen. (**A**) The color map presents the positive lesion in the peripheral zone (white arrow), while the clinically significant cancer is negative in prostatectomy specimen (false positive). (**B**) Clinically significant prostate cancer is positive in prostatectomy specimen (black arrow), while the color map is negative (false negative).

Preoperative MRI using the ADC-based bi-color map identified 215 positive lesions. Among these lesions, 133 had been categorized as PI-RADS score ≥ 3 on preoperative MRI, whereas 82 lesions had not been scored as PI-RADS score ≥ 3 before surgery. A total of 265 csPC lesions were identified in radical prostatectomy specimens, of which 126 lesions (47.5%) corresponded to ADC-based bi-color map-positive lesions. Therefore, 139 csPC lesions (52.5%) were not detected by the ADC-based bi-color map. Figure 4 shows representative examples of false-positive and false-negative cases in prostatectomy specimens. Among 215 ADC-based bi-color map-positive lesions, csPC was pathologically confirmed in 126 lesions (58.6%). Notably, among 82 ADC-based bi-color map-positive lesions that had not been categorized as PI-RADS score 3–5 on preoperative MRI, csPC was identified in 55 lesions (67.1%). In contrast, among 133 lesions categorized as PI-RADS score 3–5 on preoperative MRI, csPC was confirmed in 71 lesions (53.4%) (Table 7). 

## 4. Discussion

In this study, we evaluated the clinical value of a novel ADC-based bi-color map for prostate MRI interpretation. The results demonstrated that the combined use of PI-RADS assessment and the ADC-based bi-color map was associated with improved risk stratification of csPC, particularly in PI-RADS score 4 lesions. However, because biopsy targets in routine clinical practice were selected primarily according to PI-RADS assessment, the present study should not be interpreted as prospective proof that the ADC-based bi-color map independently improves overall cancer detection. Rather, our findings suggest that the ADC-based bi-color map may serve as a supplementary lesion-highlighting and risk-stratification tool for MRI-visible lesions.

Color-coded visualization techniques are increasingly used in medical imaging to facilitate lesion interpretation and improve visual conspicuity [20]. Color scales include grayscale and hot iron scales with a linear increase in luminance, as well as rainbow-like scales characterized by hue variations [21,22]. Zabala-Travers et al. investigated whether the choice of color scale affects readers’ ability to identify the lowest ADC value within the tumors. Although they demonstrated no significant difference among color scale types in identifying the location of the minimum ADC value [20], delineation of suspicious lesion boundaries still depends largely on subjective interpretation. In prostate MRI, accurate delineation of suspicious lesions is particularly important for biopsy targeting. Our ADC-based bi-color map was designed to simplify lesion visualization by applying predefined ADC thresholds using two distinct colors. Because the present approach is based on quantitative ADC thresholding rather than subjective visual assessment alone, it may help standardize lesion visualization and potentially reduce interpretive variability, although this hypothesis was not directly evaluated in the present study.

The potential confounding impact of the T2 shine-through effect was not fully investigated in this study. Because the ADC-based bi-color map relies on absolute ADC thresholds, benign conditions with prolonged T2 relaxation times, such as edema or cystic changes, could potentially manifest as false-positive lesions. Although the T2 shine-through effect may affect visual assessment of high b-value diffusion-weighted imaging, the present study was based on quantitative ADC map thresholding rather than qualitative interpretation of DWI signal intensity. Therefore, the influence of T2 shine-through on the present ADC-based approach is considered limited.

Formal diagnostic performance analyses demonstrated that the ADC-based bi-color map had moderate sensitivity and specificity for csPC detection in the biopsy cohort. The observed positive predictive value and negative predictive value suggest that the ADC-based bi-color map may be most useful as a complementary tool for lesion risk stratification rather than as a stand-alone diagnostic method. Subgroup analyses according to PI-RADS category demonstrated that ADC-based bi-color map positivity was significantly associated with csPC in PI-RADS score 4 lesions, whereas additional value in PI-RADS score 3 and PI-RADS score 5 lesions was limited. The relatively limited impact in PI-RADS score 5 lesions may reflect the already high baseline probability of csPC in these lesions. These findings are consistent with previous reports demonstrating that quantitative ADC parameters may improve risk stratification of equivocal MRI lesions [18,19].

The prostatectomy cohort provided additional radiologic–pathologic correlation regarding ADC-based bi-color map-positive lesions that were not categorized as PI-RADS score ≥ 3 on preoperative MRI. In this cohort, 55 of 82 ADC-based bi-color map-positive lesions outside PI-RADS score ≥3 lesions corresponded to csPC foci in prostatectomy specimens. These findings suggest that the ADC-based bi-color map may identify additional suspicious lesions that could be overlooked during routine MRI interpretation. However, pathological examination also demonstrated a substantial number of false-negative lesions, with only 126 of 265 pathological csPC foci corresponding to ADC-based bi-color map-positive lesions. Therefore, the ADC-based bi-color map should not be interpreted as a comprehensive lesion detection method intended to identify all clinically significant lesions. Small pathological tumor foci, limited MRI spatial resolution, and biological heterogeneity of csPC may all contribute to false-negative findings.

An important methodological issue in the present study is MRI acquisition heterogeneity. Absolute ADC values are known to vary across MRI scanners, vendors, field strengths, acquisition protocols, and institutions. In this retrospective study, fixed ADC thresholds were applied universally without scanner-specific normalization, harmonization, or phantom-based calibration procedures. Subgroup analyses demonstrated that the ADC-based bi-color map maintained significant associations with csPC in both internally and externally acquired MRI examinations, although diagnostic performance characteristics differed somewhat between the groups. MRI examinations performed at our institution showed relatively higher sensitivity, whereas externally acquired MRI examinations demonstrated relatively higher specificity. These findings suggest that scanner-dependent variability may influence the behavior of fixed ADC threshold-based approaches. Although inclusion of heterogeneous MRI examinations may reflect real-world clinical practice, the robustness and transportability of the present method across imaging platforms remain uncertain. Future multicenter studies incorporating scanner-specific normalization, phantom calibration, or platform-stratified validation are warranted.

The predefined ADC thresholds used in the present study were derived from our previous research and were not optimized using the current dataset. Although this strategy avoided post hoc threshold optimization, the generalizability and stability of these thresholds across different prostate zones and MRI acquisition protocols remain uncertain. Additional prospective studies are needed to determine whether alternative or zone-specific ADC thresholds may further improve diagnostic performance.

The present study also highlights broader translational challenges associated with quantitative imaging tools. Although the ADC-based bi-color map is conceptually simple and does not rely on deep learning algorithms, successful clinical implementation still requires standardized evaluation protocols, standardized image acquisition, reproducibility across scanners and institutions, transparent reporting of diagnostic performance, external validation, and demonstration of meaningful clinical utility [23]. Therefore, the present ADC-based bi-color map should currently be regarded as a preliminary decision-support and lesion-highlighting tool requiring prospective multicenter validation before routine clinical implementation.

Several limitations should be acknowledged. First, this was a retrospective study with a relatively limited sample size. Second, analyses were primarily performed at the lesion level, although several patients contributed more than one lesion. Therefore, complete statistical independence may not have been fully satisfied, and within-patient clustering may have resulted in underestimated uncertainty or inflation of statistical significance. Although lesion-level analysis was intentionally adopted because the primary objective was radiologic–pathologic lesion correspondence, future studies should consider mixed-effects modeling or generalized estimating equation approaches. Third, reader experience, interobserver agreement, and the influence of the ADC-based bi-color map on reader performance were not evaluated. Therefore, any potential reduction in interobserver variability remains hypothetical. Finally, despite improved risk stratification in PI-RADS score 4 lesions, false-positive and false-negative findings remained. Accordingly, the ADC-based bi-color map should be interpreted as an adjunctive imaging tool rather than a replacement for conventional multiparametric MRI assessment and clinical judgment.

## 5. Conclusions

This study demonstrated that the combined use of PI-RADS assessment and a novel ADC-based bi-color map was associated with improved risk stratification of csPC, particularly in PI-RADS 4 lesions. In addition, the ADC-based bi-color map identified suspicious lesions that were not categorized as PI-RADS ≥ 3 on conventional MRI assessment, suggesting a potential role as a supplementary lesion-highlighting tool.

## Figures and Tables

**Table 1:** Patients’ characteristics and target lesion’s characteristics.

The MRI-Transrectal Ultrasound Fusion-Guided Biopsy Group	
No. of Patients	108
Age (Year), IQR	69 (63–74)
PSA (ng/mL), IQR	7.70 (6.13–11.10)
PI-RADS Score (%)	
3	20 (18.5%)
4	71 (65.8%)
5	17 (15.7%)
Targeted Biopsy (lesion)	157
The Robot-Assisted Radical Prostatectomy Group	
No. of Patients	93
Age (Year), IQR	68 (63–71.0)
PSA (ng/mL), IQR	7.7 (5.45–13.0)
Pathological T Stage (%)	
T2a	10 (10.8%)
T2b	2 (2.2%)
T2c	55 (59.1%)
T3a	19 (20.4%)
T3b	7 (7.5%)
ISUP Grade Group at Surgical Specimen (%)	
Grade Group 1	2 (2.2%)
Grade Group 2	34 (36.6%)
Grade Group 3	35 (37.6%)
Grade Group 4	11 (11.8%)
Grade Group 5	11 (11.8%)

**Table 2:** Positive rates of clinically significant prostate cancer according to PI-RADS score in the MRI-transrectal ultrasound (TRUS) fusion-guided biopsy group.

	*n*	Significant Cancer	Benign or Insignificant Cancer	*p* Value
total	157	65 (41.4%)	92 (58.6%)	
PI-RADS score 3	33	5 (15.2%)	28 (84.8%)	
PI-RADS score 4	103	43 (41.7%)	60 (58.3%)	
PI-RADS score 5	21	17 (81.0%)	4 (19.0%)	<0.001

**Table 3:** Association Between PI-RADS Score and ADC-based bi-color map in the MRI-transrectal ultrasound (TRUS) fusion-guided biopsy group.

	*n*	Color Map Positive	Color Map Negative	*p* Value
total	157	70 (44.6%)	87 (55.4%)	
PI-RADS score 3	33	7 (21.2%)	26 (78.8%)	
PI-RADS score 4	103	46 (44.7%)	57 (55.3%)	
PI-RADS score 5	21	17 (81.0%)	4 (19.0%)	<0.001

**Table 4:** Positive rates of clinically significant prostate cancer according to ADC-based bi-color map in the MRI-transrectal ultrasound (TRUS) fusion-guided biopsy group.

		Significant Cancer	Benign or Insignificant Cancer	*p* Value
ADC-based bi-color map	positive	49 (70.0%)	21 (30.0%)	
	negative	16 (18.4%)	71 (81.6%)	<0.001
**Parameter**		**Value (%)**	**95% CI (%)**	
Sensitivity		75.4	63.1–85.2	
Specificity		77.2	67.2–85.3	
Positive Predictive Value		70.0	57.9–80.4	
Negative Predictive Value		81.6	71.9–89.1	
Accuracy		76.4	69.0–82.8	

**Table 5:** Biopsy results stratified by PI-RADS score and ADC-based bi-color map in the MRI-transrectal ultrasound (TRUS) fusion-guided biopsy group.

			Significant Cancer	Benign or Insignificant Cancer	*p* Value
PI-RADS score 3	Color map	positive	2 (40%)	5 (17.9%)	
		negative	3 (60%)	23 (82.1%)	0.282
PI-RADS score 4	Color map	positive	32 (74.4%)	14 (23.3%)	
		negative	11 (25.6%)	46 (76.7%)	<0.001
PI-RADS score 5	Color map	positive	15 (88.2%)	2 (50%)	
		negative	2 (11.8%)	2 (50%)	0.148

**Table 6:** Association between ADC-based bi-color map status and clinically significant prostate cancer according to MRI acquisition site.

		MRI at Our Institution (*n* = 63)			MRI at External Institutions (*n* = 94)		
		Significant Cancer	Benign or Insignificant Cancer	*p* Value	Significant Cancer	benign or Insignificant Cancer	*p* Value
ADC-based bi-color map	positive	31	10		18	11	
	negative	8	14		8	57	
	total	39	24	0.002	26	68	<0.001

**Table 7:** Association between ADC-based bi-color map and radical prostatectomy specimens.

		Prostatectomy Specimens	
	*n*	Significant Cancer Positive	Significant Cancer Negative
Color map-positive lesions	215	126 (58.6%)	89 (41.4%)
Lesions scored as PIRADS 3/4/5 on preoperative-MRI	133	71 (53.4%)	62 (46.6%)
Lesions not scored as PIRADS 3/4/5 on preoperative-MRI	82	55 (67.1%)	27 (32.9%)

## Data Availability

The data that support the findings of this study are available from the corresponding author upon reasonable request. The data are not publicly available due to privacy or ethical restrictions.

## Abbreviations

PC	Prostate cancer
MRI	Magnetic resonance imaging
PI-RADS	Prostate Imaging-reporting and Data System
csPC	clinically significant prostate cancer
ADC	Apparent Diffusion Coefficient
TRUS	Transrectal ultrasound
RARP	Robot-assisted radical prostatectomy
ISUP	International Society of Urological Pathology
PSA	Prostate-specific antigen
